# Molecular Morphology and Viscoelasticity of ASP Solution under the Action of a Different Medium Injection Tool

**DOI:** 10.3390/polym11081299

**Published:** 2019-08-02

**Authors:** Bin Huang, Xinyu Hu, Cheng Fu, Haoran Cheng, Xin Wang, Li Wang

**Affiliations:** 1 Key Laboratory of Enhanced Oil Recovery, Ministry of Education, College of Petroleum Engineering, Northeast Petroleum University, Daqing 163318, China; 2 Post-Doctoral Scientific Research Station, Daqing Oilfield Company, Daqing 163413, China; 3 Research Institute of Tsinghua University in Shenzhen, Shenzhen 518057, China; 4 ICORE GROUP INC, Shenzhen 518057, China; 5 Research Institute of Production Engineering, Daqing Oilfield, Daqing 163453, China

**Keywords:** ASP solution, different medium injection tool, molecular micromorphology, static shear experiment, dynamic mechanical experiment

## Abstract

In order to improve the oil displacement effect of alkali/surfactant/polymer (ASP) solution in low-permeability oil layers, Daqing Oilfield has proposed a separate injection technology. The objective of separate injection technology is to reduce the viscosity of ASP solution through a different medium injection tool and increase the injection amount of ASP solution in low permeability oil layer, thus improving the oil displacement effect. In order to study the effect of the different medium injection tool on ASP solution, SEM is used to observe the changes in molecular micromorphology before and after the ASP solution flows through the tool. Then, the influence of the tool on viscosity and the first normal stress difference of the solution are studied through static shear experiments. Finally, the storage and loss modulus of the solution are measured through dynamic mechanical experiments and the relaxation time and zero shear viscosity of the solution are verified and compared. The results show that molecular chains are obviously broken and the grid structure is destroyed after the ASP solution is acted on by the different medium injection tool. The viscosity and elasticity of ASP solution decrease, and the influence degree of the different medium injection tool on viscosity is greater than elasticity. The results of the steady shear experiment and dynamic mechanics experiment are consistent. Therefore, the different medium injection tool can achieve the purpose of use, which is conducive to the injection of displacement fluid into low-permeability oil layers and enhance the recovery ratio.

## 1. Introduction

During the continuous exploitation of crude oil in oil fields, alkali/surfactant/polymer (ASP) flooding is a method of greatly improving the oil recovery rate. Under the synergistic effect of alkali, surfactant, and polymer, the use of ASP solution for oil displacement can reduce the oil-water interfacial tension and fluidity ratio to improve solution viscosity and sweep efficiency and give full play to the oil displacement effect [1,2,3,4,5,6]. ASP flooding technology can effectively improve oil recovery under the condition of extremely high water cut in an oil field [7,8,9]. However, due to the heterogeneity of oil layers, high-viscosity ASP solution has a large injection amount in high-permeability oil layers, but will cause blockage in low-permeability oil layers, thus reducing the injection amount. If low-viscosity ASP solution is used, the oil displacement efficiency in high-permeability oil layers is lower [10,11]. Therefore, in order to solve the problem of easy plugging of injection displacement fluid in low-permeability reservoirs, Daqing Oilfield proposes a separate injection technology for ASP solution. The working principle is to use a partial pressure tool to reduce injection pressure and control injection volume in high-permeability reservoirs. In low-permeability oil layers, a different medium injection tool is used, and mechanical shearing is used to reduce the viscosity of the ASP solution, so that the solution can be injected into the oil layer, improving the overall oil displacement efficiency.

Huang et al. [10] conducted core flooding experiments with different polymer molecular weights to determine the matching relationship between polymer molecular weights and reservoir permeability in ASP flooding. The results indicate that when the ratio of the pore throat radius (rh) to the polymer molecular cyclotron radius (rp) is greater than 7, the injection of ASP system with a variety of molecular weights will not be blocked; on contrary, when the ratio is less than 7, the core will be blocked. Seright et al. [12] studied the influence of three main EOR polymer properties on injection performance, and quantitatively studied the rheology and mechanical degradation of polymers. The results indicate that increased injectant viscosity could substantially reduce injectivity, slow fluid throughput, and delay oil production from flooded patterns. Luo et al. [13] studied the matching relationship between polymer molecular size and pore size in Karamay conglomerate reservoir of Xinjiang oilfield. The molecular size of polymer was studied by nuclear pore membrane filtration method. The results show that polymer DQ3500 with molecular weight of 3.57 × 10^7^- and 0.7-mm microporous membrane match well. Zhao et al. [14] studied that the molecular weight of polymer has an important effect on polymer flooding efficiency. Based on laboratory experiments and numerical simulation, two matching maps were formed and used to optimize the molecular weight of polymer flooding. The method has been applied to the secondary oil recovery project in Daqing Oilfield, and the recovery ratio has been increased by more than 8%. Hu et al. [15] studied the maximum allowable molecular weight of polymer and the concentration under given permeability through core flow experiments. The results show that too high viscosity and molecules will block the low permeability layer. Polymer molecules are spherical and the ratio of average diameter to average pore throat should be greater than 5 or 10 to avoid blockage. The above research shows that the molecular weight of the polymer solution has a direct influence on the oil displacement effect of low permeability reservoirs, and the viscosity of the solution is related to the molecular weight [16]. Therefore, lowering the viscosity of the polymer solution can improve the oil displacement effect of the displacement agent in low permeability reservoirs.

For the Daqing oil fields with serious formation heterogeneity, the general injection method will result in low sweep degree of medium and low permeability oil layers, which will seriously affect the development effect of the oil fields. Daqing oil fields proposed separate injection technology can control the injection amount of displacement fluid in high permeability oil layers, increase the injection amount of medium and low permeability oil layers, and improve the overall displacement effect. Researchers have carried out the following related studies on separate injection technology. Chen and Duan [17], through laboratory and field tests, studied polymer single-pipe multilayer separate injection technology and gave the design principle of separate injection tools. Geng et al. [18] studied the structure, process characteristics, and adaptability of intermittent injection, annular depressurization groove, and concentric injection string with slender pipe, and put forward the research idea of eccentric partial pressure and mass injection technology. Zhou et al. [19] solved the problem of large injection pressure difference between high- and low-permeability layers by using an eccentric double-layer packer, injection distributor, and surface pressure regulator. Li et al. [20] proposed a technology consisting of concentric, eccentric, and stratified separate injection, and put forward reasonable separate injection timing and principles. The above-mentioned research mainly focused on partial pressure technology, in which the injection pressure is reduced and the injection quantity is controlled in high-permeability oil layers. However, there is little research on improving the injection ability of a displacement agent in low permeability oil layer to improve the oil displacement effect.

Based on the technique of separate injection and the theory of solution shear degradation [21,22,23,24,25], the concept of the different medium injection tool is proposed, that is, a tool for active shearing of solution to solve the injection problem of a displacement agent in a low permeability oil layer [26,27,28,29] Huang et al. [30] established a rheological model of polymer solution in the different medium injection tool by combining the rheological characteristics of polymer and local perturbation theory, and determined the maximum injection speed. Chen et al. [31] and Huang et al. [32] analyzed the flow characteristics of ASP solution in the different medium injection tool by finite element analysis. The research shows that with increased diameter of the flow channel of the tool, the viscosity loss and velocity of ASP solution decrease, and more nozzle stages leads to greater pressure drop and viscosity loss of ASP solution. Zhang et al. [33] analyzed the influence of the structure of the different medium injection tool on average strain rate and pressure loss through a combination of numerical simulation and orthogonal experiment to determine the optimal structural parameters of the tool. That research provides theoretical support and mathematical research methods for the experimental study of rheological properties of ASP solution in the different medium injection tool, however, there is a lack of research on laboratory tests and field experiments, and the reliability of theoretical results is poor.

The above-mentioned studies have little research on improving the oil displacement effect of low-permeability reservoirs. The implementation of separate injection technology by using the different medium injection tool to improve the contradiction between reservoirs is only at the stage of theoretical research and numerical simulation and cannot actually simulate the field situation. In addition, most of the experiments and simulations take polymer solution as the research object and there is less research on ASP solution. And most of the experimental research methods for polymer and other displacement agents are mainly aimed at analyzing the characteristic structure of molecular micromorphology [34,35,36,37] and the relevant parameters of shear rheological experiments [38,39,40,41,42,43,44,45,46,47,48]. Such research methods are relatively single. Therefore, it is of great significance for the reliability and integrity of experimental results to study the micromorphology and rheological properties of ASP solution under the action of different medium injection tools from the microscopic and macroscopic perspectives and to carry out mutual verification of experiments.

In this paper, ASP solution with molecular weights of 16 million, 19 million, and 25 million and concentrations of 1000 mg/L and 2000 mg/L is taken as the research object. Scanning electron microscopy (SEM) is used to study the changes in molecular morphology and molecular structure of ASP solution with different molecular weights and concentrations before and after the action of the different medium injection tool. The changes in viscosity and the first normal stress difference are studied through rheological steady shear experiments. Finally, the changes in storage modulus and loss modulus are studied through dynamic mechanical experiments, the degree of influence of viscosity and elasticity is analyzed, and the results obtained from the steady shear experiment are verified.

## 2. Materials and Methods

### 2.1. Model of the Different Medium Tool

**Figure 1:**
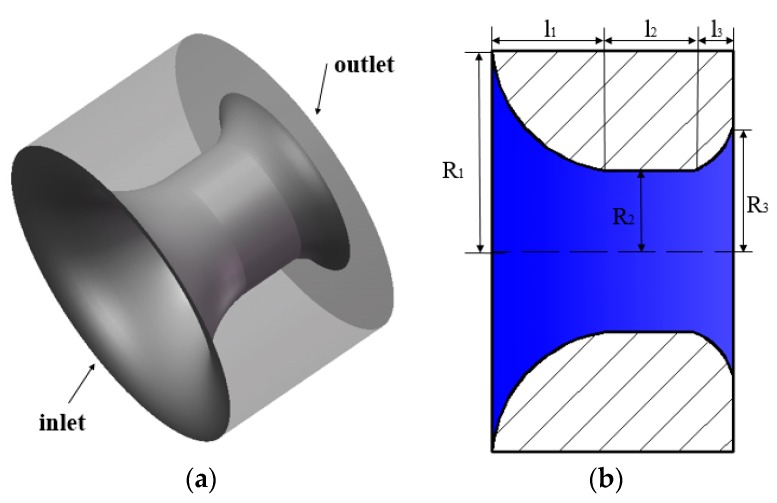
Structure of the different medium tool: (**a**) three-dimensional model, (**b**) two-dimensional structural model.

The different medium tool used in the experiment is made of 316 L stainless steel (Daqing Oil Production Research Institute, Daqing, China). The surface of the tool is smooth, and the surface of the channel must be polished. Figure 1a shows a 3-dimensional model of the different medium tool.

Figure 1b shows a 2-dimensional structural model of the tool, which is mainly divided into contraction, cylinder, and diffusion sections. The middle part of the tool is the region where the solution flows through. The flow of ASP solution in the tool can be seen as a flow process that first contracts and then diffuses in a pipeline with variable cross-sections. Under the action of the contraction and diffusion sections, ASP solution is actively sheared to achieve the purpose of different medium injection. Typical structural parameters of the different medium tool are shown in Table 1.

### 2.2. Chemicals

The polymer used to prepare ASP solution in this experiment is partially hydrolyzed polyacrylamide (HPAM), with relative molecular weights of 16 million, 19 million, and 25 million and a hydrolysis degree of about 26% (Daqing Oilfield Production Technology Research Institute, Daqing, China). The surfactant is ORS-41 (alkylbenzene sulfonate) with an effective content of 50% (Daqing Petroleum Refining and Chemical Company of China, Daqing, China). The alkali is NaOH with a concentration of 30% (Daqing Oilfield Oil Production Engineering Research Institute, Daqing, China).

### 2.3. Brine

Under the actual production conditions of an oil field, because the formation water is not pure water, in order to simulate the oil displacement effect of ASP solution in the formation more realistically, brine is selected as a diluent to prepare the solution (No.1 Oil Production Plant of Daqing Oilfield, Daqing, China). The composition of the brine is shown in Table 2. A filter with a filter membrane is required before use.

### 2.4. Experimental Procedure

#### 2.4.1. Solution Preparation

A YP-B2003 electronic balance (Shanghai Guangzheng Medical Instrument Co., Ltd, Shanghai, China) is used to measure the dry powder of polymers with different molecular weights and experimental water (brine) in proportion. The mixture is put into a glass beaker and dissolved and stirred with a EURO-ST D S25 electronic stirrer (IKA, Staufen, Germany) at 250 r/min for 2.5 h to form a transparent aqueous solution, which is then put into an HW-III thermostat (provided by Hai’an Huada Petroleum Instrument Co., Ltd, Jiangsu, China) for constant temperature treatment. The weighed surfactant and alkali are added to the polymer solution, and the mixture is continuously stirred in an electronic stirrer at a rotating speed of 400 r/min for 30 min. Then, a certain amount of experimental water (brine) is added to dilute the ASP solution, and ASP solution with molecular weights of 16 million, 19 million, and 25 million and concentrations of 1000 mg/L and 2000 mg/L are obtained according to the solution concentration experimental scheme (Daqing Oilfield Co., Ltd, Daqing, China). The prepared solution is allowed to stand for 6 h before use to ensure full dispersion of molecules and uniformity.

#### 2.4.2. Experimental Study

**Figure 2:**
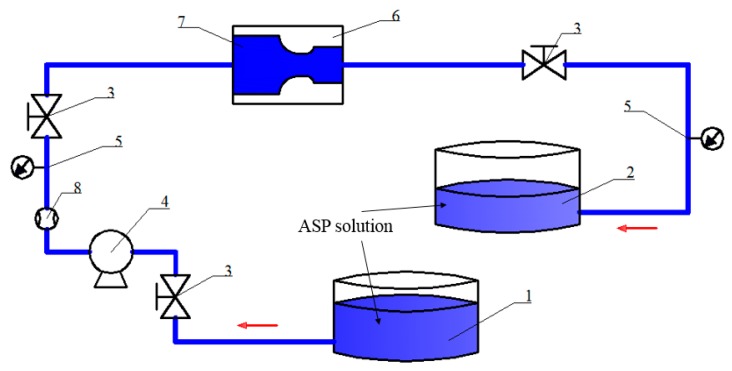
Technological process: (1) stirrable liquid supply tank; (2) return liquid tank; (3) valve; (4) screw pump; (5) pressure gauge; (6) flow simulation chamber; (7) different medium tool; (8) flow meter.

As shown in Figure 2, the prepared ASP solution with different molecular weights and concentrations is put into the stirrable liquid supply tank in batches, the heat preservation system is used to keep the temperature of the solution at 45 °C, after checking the connection tightness of each facility is good the valve is opened to start the liquid supply pump, the flow rate is controlled at 50 m^3^/d, the solution flows through the different medium tool (provided by Daqing Oil Production Research Institute, Daqing, China, the structural parameters are shown in Table 1) and back to the return liquid tank, and then the ASP solution in the stirrable liquid supply tank and the return liquid tank is sampled for detection.

The micromolecular structure experiment is conducted by transferring the treated ASP solution to a freezing table and smearing it on the upper surface of a designated solid, then quickly pouring liquid nitrogen to freeze it at −70 °C, the freezing time lasts for about 10 min, and placing the frozen sample into an A1930500 vacuum freeze dryer (Shanghai Ice River Electronic Technology Co., Ltd, Shanghai, China) to sublimate the water in the sample and obtain the final dry sample. The prepared dry sample is placed in a high-voltage electric field with a certain vacuum degree to ionize the air, and then a layer of conductive metal (gold) film is plated on the surface of the dry sample, and gold spraying is carried out twice for 30 s each time. The sample is quickly transferred to the observation table in the sample room of a Quanta 450 FEG scanning electron microscope (FEI, Hillsboro, OR, USA) for observation, photos of typical areas of the sample are taken, and relevant sample structure analysis is carried out.

The viscoelastic experimental study of ASP solution before and after the action of the different medium tool is conducted by starting the constant temperature circulation system of an RS-150 rheometer (HAAKE, Staufen, Germany) and heating it to the measured temperature 45 °C for 15 min. The ASP solution is put into the preheated measuring outer cylinder and keep the temperature 45 °C constant for 20 min, so that each point of the sample can reach the testing temperature uniformly. The constant shear rate CR mode is selected, and the shear rate tested is set to be 5–1500 s^−1^. The rheometer is started to rotate the rotor, and the viscosity and the first normal stress difference are detected in a stable measurement mode. Then dynamic measurement is adopted, setting the frequency at 0.01–10 Hz (in linear viscoelasticity), scanning the frequency at 0.1 Pa, and measuring the storage and loss modulus in the stress scanning range of 0.01–50 Pa. When the indication value is basically stable, start recording, and then record every 5 min. If the deviation between the three calculated values of the four continuously recorded values and the first one is not more than 5%, the system is considered to have reached the dynamic equilibrium value.

ASP solutions of each molecular weight and concentration were measured three times, and the measured results were averaged.

### 2.5. Experimental Theory

#### 2.5.1. Viscosity

**Figure 3:**
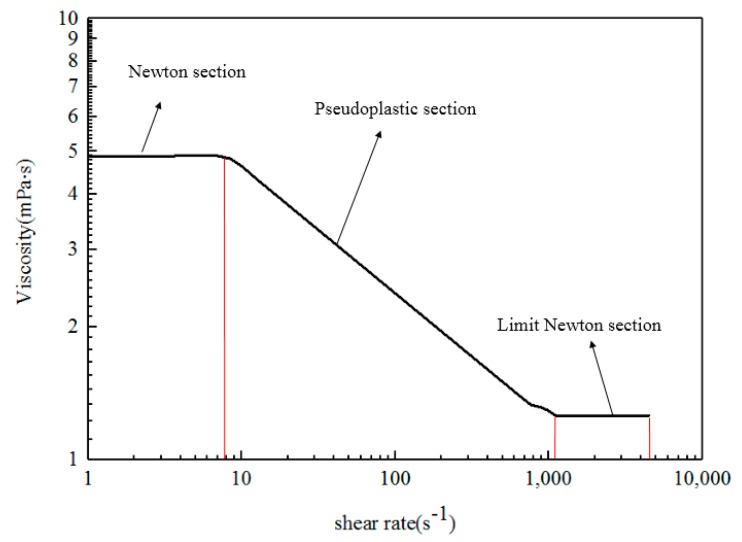
Rheological curve.

The ASP solution is a non-Newtonian fluid. At low shear rate, it generally shows the characteristics of Newtonian fluid. At this time, the viscosity is a constant. With increased shear rate, the viscosity of the fluid decreases, showing the characteristics of shear thinning. When the shear rate increases to a certain value, the viscosity tends to reach a second stable value [49]. The complete rheological curve is shown in Figure 3.

In the region of high shear rate and low shear rate, the Carreau model is suitable for all shear regions:

(1) $$\eta (\dot{\gamma })=\eta_{\infty }+(\eta_{o}-\eta_{\infty }){\left[1+{\left(\lambda \dot{\gamma }\right)}^{2}\right]}^{\frac{n-1}{2}}$$

where $\eta_{0}$ and $\eta_{\infty }$ respectively represent the viscosity values under the conditions of zero shear viscosity and high shear rate, and can reflect the characteristic that the viscosity of the solution is constant under specific conditions; And λ is relaxation time, or called a characteristic time constant; and n is a power law index, which indicates the degree to which the flow characteristics deviate from Newtonian fluid.

#### 2.5.2. First Normal Stress Difference

In the process of ASP solution flow, the total stress on the internal volume element of the solution is expressed by tensors as follows:

(2) $$\tau =\left[\begin{array}{ccc} \tau_{xx} & \tau_{xy} & \tau_{xz}\\ \tau_{yx} & \tau_{yy} & \tau_{yz}\\ \tau_{zx} & \tau_{zy} & \tau_{zz} \end{array}\right]$$

where $\tau_{xx}$, $\tau_{yy}$, and $\tau_{zz}$ are normal stresses, the others are shear stresses, and $\tau_{xy}=\tau_{yx}$, $\tau_{xz}=\tau_{zx}$, $\tau_{yz}=\tau_{zy}$, and the first normal stress difference $N_{1}$ is defined as the difference between the stresses in the flow direction and the velocity gradient direction. Then the first normal stress difference is expressed as

(3) $$\tau_{xx}-\tau_{yy}=N_{1}(\overset{\cdot }{\gamma })$$

In the macromolecule system, the chain molecules are approximately a spherical envelope volume in the static state, and become ellipsoid in the flow field. Restoring force will be generated in these deformed microstructures. As these structures are anisotropic, the restoring force generated is also anisotropic, and the main axis of the ellipsoid after deformation tends toward the flow direction. Therefore, the restoring force in this direction is greater than that in the other two vertical directions, and these restoring forces generate normal stress components.

The first normal stress difference is the main characteristic of viscoelastic fluid [41], which can generate axial pressure. The first normal stress difference of viscoelastic polymer solution comprehensively reflects the force of elasticity of polymer solution on crude oil. In the study, the first normal stress difference is taken as a parameter to characterize the elasticity of polymer solution.

#### 2.5.3. Storage Modulus and Loss Modulus

In the dynamic mechanics experiment, if a harmonic stress (or strain) is applied to the solution, the stress (or strain) of the solution also changes with time in a harmonic law. The stress (or strain) is a function of time, and its modulus is usually expressed by a complex modulus. The strain change of viscoelastic solution lags behind the stress change by a phase angle. For the latter, the stress change leads the strain by a phase angle, then the change of strain and stress of ASP solution with time can be expressed as follows:

(4) $$\gamma \left(t\right)=\gamma_{0}\sin\omega t$$

(5) $$\tau \left(t\right)=\tau_{0}\sin(\omega t+\delta )$$

According to the definition of modulus, two different moduli can be obtained. $G^{\prime }$ is defined as the ratio of stress and strain in the same phase, and $G^{\prime \prime }$ is the ratio of stress and strain amplitude when the phase difference is $\frac{\pi }{2}$, namely

(6) $$G^{\prime }=\left(\tau_{0}/\gamma_{0}\right)\cos\delta$$

(7) $$G^{\prime \prime }=\left(\tau_{0}/\gamma_{0}\right)\sin\delta$$

Equation (5) can be changed to

(8) $$\tau \left(t\right)=\gamma_{0}G^{\prime }\sin\omega t+\gamma_{0}G^{\prime \prime }\cos\omega t$$

The above equation is converted into a complex form, called complex modulus $G^{*}$, where

(9) $$G^{*}=G^{\prime }+iG^{\prime \prime }$$

In the formula, $G^{\prime }$ is the real modulus or can be called the storage modulus, which represents the energy stored by ASP solution due to elastic deformation and can be used to characterize the elastic characteristics of the fluid. $G^{\prime \prime }$ is an imaginary modulus, which can also be called a loss modulus, and represents the energy of ASP solution due to heat loss during deformation. The energy used in this part of the flow is irreversible loss converted into shear heat and can be used to characterize the viscosity of the fluid.

According to the constitutive equation of Maxwell’s model:

(10) $$\tau +\lambda \frac{d\tau }{dt}=G\lambda \frac{d\gamma }{dt}$$

where λ is relaxation time and G is stress relaxation modulus, and $\lambda =\frac{\eta }{G}$.

Stress and strain are expressed as circular vibration functions:

(11) $$\gamma =\gamma_{o}e^{i\omega t}$$

(12) $$\tau =\tau_{o}e^{i(\omega t+\delta )}$$

According to the Equation (9), it can be obtained:

(13) $$G^{*}=G\frac{i\omega \lambda }{1+i\omega \lambda }$$

Therefore, the storage modulus and loss modulus of Maxwell model are:

(14) $$G^{\prime }=G\frac{\omega^{2}\lambda^{2}}{1+\omega^{2}\lambda^{2}}$$

(15) $$G^{\prime \prime }=G\frac{\omega \lambda }{1+\omega^{2}\lambda^{2}}$$

## 3. Results and Discussion

### 3.1. Effect of the Different Medium Tool on Molecular Micromorphology of ASP Solution

**Figure 4:**
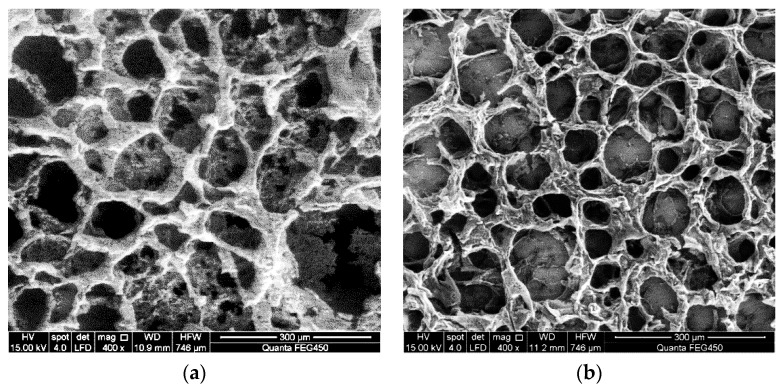

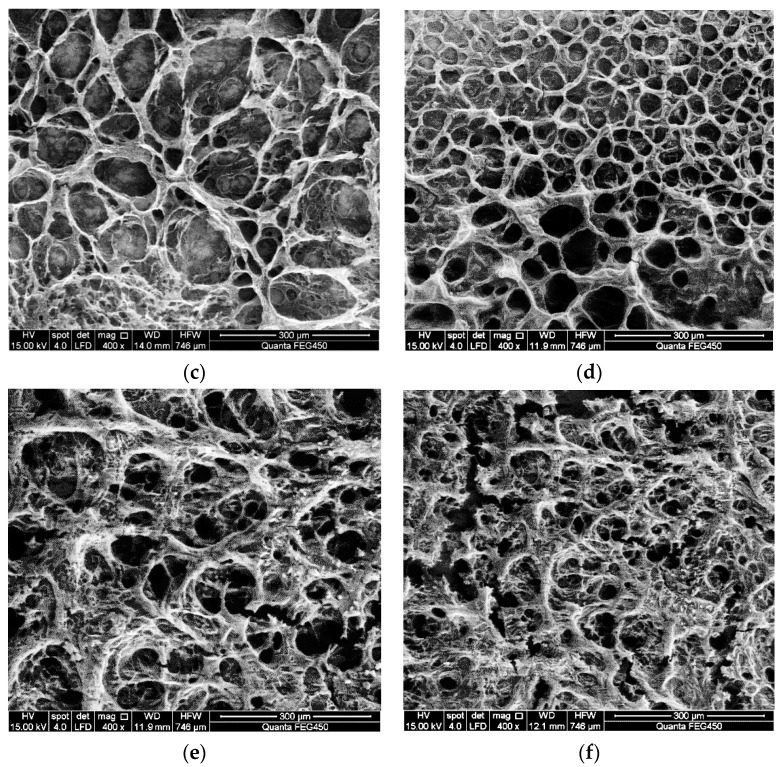
Molecular morphology of alkali/surfactant/polymer (ASP) solution: (**a**) 16 million (MD), 1000 mg/L; (**b**) 16 million (MD), 2000 mg/L; (**c**) 19 million (MD), 1000 mg/L; (**d**) 19 million (MD), 2000 mg/L; (**e**) 25 million (MD), 1000 mg/L; (**f**) 25 million (MD), 2000 mg/L.

Scanning electron microscopy is used to observe the molecular micromorphology of ASP solution with molecular weights of 16 million, 19 million, and 25 million and concentrations of 1000 mg/L and 2000 mg/L before flowing through the different medium tool, as shown in Figure 4.

As shown in Figure 4, ASP solution prepared by polymers with different concentrations of molecular weight under the same molecular weight has similar spatial structure of microscopic morphology, generally a multilayer three-dimensional network structure with uniform size. Most of the network structures are round or nearly hexagonal meshes, and some areas show a sheet-net structure, with thick trunks and twigs connecting the meshes. This is because after HPAM is partially hydrolyzed, carboxyl anions exist on the macromolecular chains, and electrostatic repulsion exists between adjacent carboxyl groups [10]. Because NaOH is added to the solution, the number of negative charges on the molecular chains increases, resulting in enhanced electrostatic repulsion, enhanced repulsion between the molecular chains, and increased stretching of the molecular chains; each molecular chain adopts a random coil conformation, and different polymer molecular chains can penetrate each other and even twine, resulting in the formation of multilayer three-dimensional network structures with holes of different sizes in the solution. The coarse trunk and subdivision branches can directly reflect the number of groups on the molecule: the more groups there are, the thicker the branches [50,51]. The higher the concentration of polymer in ASP solution, the denser the spatial network structure and the coarser the molecular framework, as can be seen from the SEM images. As the molecular weight of the polymer increases at the same concentration, the molecular structure of ASP solution with high molecular weight is coarser and the spatial network structure is denser [15,51,52,53]. The microstructure morphology of ASP solution prepared by polymer with a molecular weight of 25 million is similar to that of ASP solution with molecular weights of 16 million and 19 million. However, the higher the molecular weight of the polymer, the closer the molecular aggregates are in the solution, and scanning electron microscope images show that more multilayer spatial structures are connected or cross-connected and the skeleton of the network structure is thicker. Although the mesh is no longer obvious in the case of high-concentration polymer, the continuous spatial network structure is more compact and complete [50,53,54,55].

**Figure 5:**
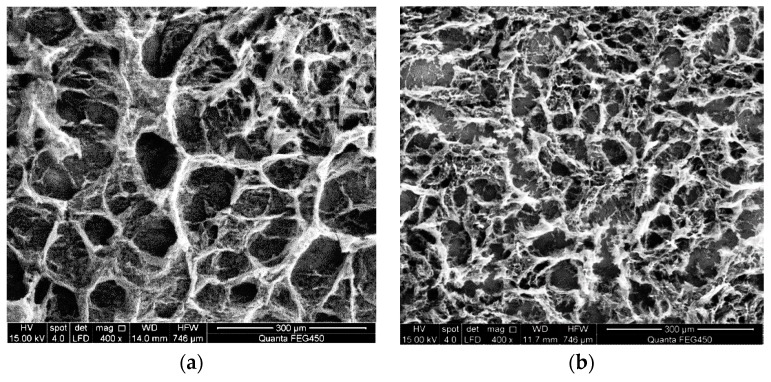

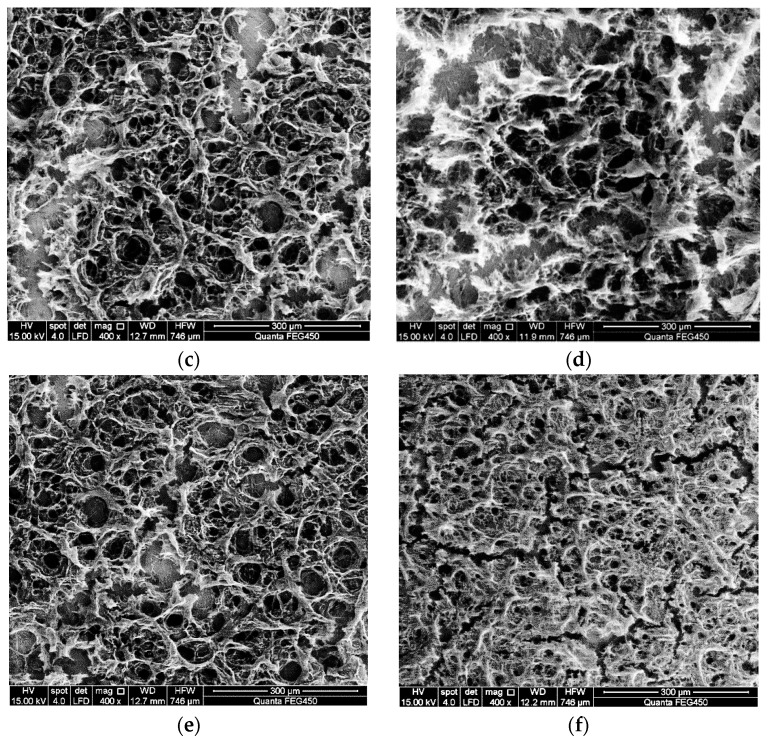
Molecular micromorphology of ASP solution after being acted on by the different medium tool: (**a**) 16 million (MD), 1000 mg/L; (**b**) 16 million (MD), 2000 mg/L; (**c**) 19 million (MD), 1000 mg/L; (**d**) 19 million (MD), 2000 mg/L; (**e**) 25 million (MD), 1000 mg/L; (**f**) 25 million (MD), 2000 mg/L.

During the action of the different medium tool, the ASP system passes through the flow channel and enters the deep part of the oil layer through the bottom hole. The flow area of the tool is small, and after ASP solution flows through the tool, the speed of the solution flowing through the contraction section changes sharply, elongating the polymer molecules. When the molecular chain is pulled to a certain length and exceeds its strength, it breaks or is degraded. The microstructure of molecules and the size of molecular coils change to some extent, which will reduce the molecular weight of the polymer and increase the viscosity loss of the solution [19,30]. The scanning electron microscope is used to observe the micromorphology of ASP solution flowing through the different medium tool, as shown in Figure 5.

As can be seen from Figure 5, due to the effect of high-speed shearing and tensile stress of the different medium tool, the solution produces a shearing degradation effect, which causes the short and long molecular chains with polar groups in ASP solution to be sheared and thus broken, the stretching property of the molecular chains becomes poor, the molecular chains are easy to curl and wind together to form an irregular spatial structure, and the original network structure becomes incomplete and the meshes become sparse. The ability of the network structure to wrap water molecules is greatly reduced, resulting in a significant decrease in the ability to increase viscosity and a macroscopic decrease in apparent viscosity of the solution [56,57,58,59,60]. The network structure of high-concentration ASP solution flowing through the different medium tool is still denser than that of low-concentration ASP solution, and the skeleton is coarser, which indicates that the rigidity of the multilayer three-dimensional network structure of high-concentration solution is better than that of low-concentration solution after the solution with different concentrations is sheared by the tool. With increased molecular weight of the polymer in ASP solution, an obvious molecular chain fracture phenomenon can be observed on electron microscope images, and the spatial network structure of the solution is more compact and continuous. Under the same concentration, the space network structure of ASP solution with a molecular weight of 25 million is the most compact. Although the molecular micromorphology space network is destroyed, some molecular chains are still intertwined, maintaining the structure of macromolecular chains, thus the network structure is more rigid. The space network of ASP solution with a molecular weight of 16 million is evacuated the most. Macroscopically, the apparent viscosity of ASP solution with a molecular weight of 25 million and a concentration of 2000 mg/L is greater, while that of ASP solution with a molecular weight of 16 million and a concentration of 1000 mg/L is the lowest after being acted on by the quality separating tool.

Comparing the molecular morphology of ASP solution with different molecular weights and concentrations before and after flowing through the different medium tool, it can be seen that the tool has an obvious effect on the solution, in that it can effectively change the molecular structure, reduce the molecular weight, and increase the viscosity loss of the solution, and it meets the design purpose.

### 3.2. Effect of the Different Medium Tool on Viscoelasticity of ASP Solution

The viscoelasticity of ASP solution is involved in preparation, injection, pipeline transportation, wellbore flow, and flow in porous media under reservoir conditions. Viscoelasticity is an important characteristic in the performance of solutions. It usually refers to the obvious viscous and elastic characteristics when multiple macromolecular motion units respond to forces [61,62,63,64,65,66]. At present, there are mainly two methods for measuring solution viscoelasticity: steady shear test and dynamic mechanical test.

**Figure 6:**
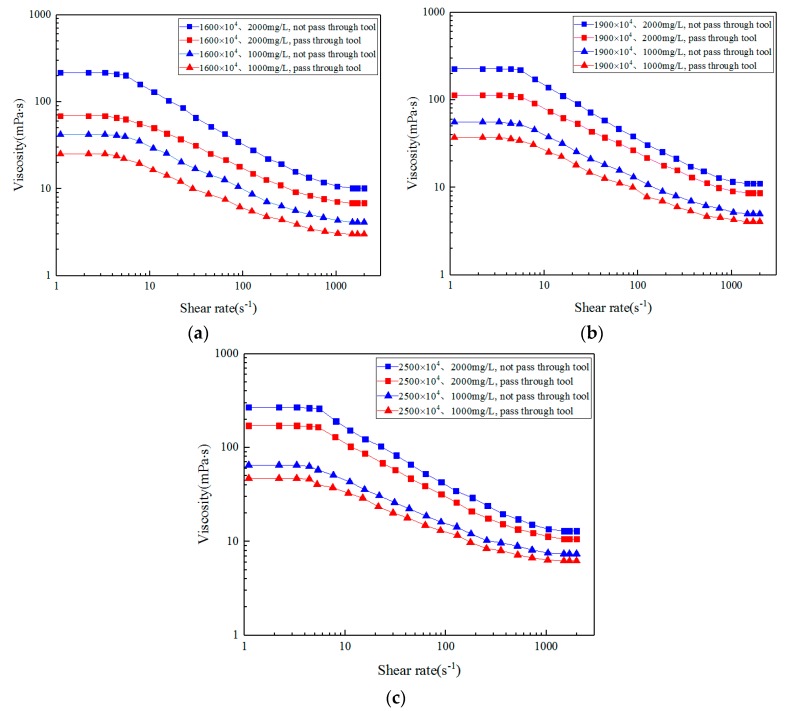
Variation of viscosity with shear rate of ASP solution before and after flowing through the different medium tool: (**a**) 16 million (MD); (**b**) 19 million (MD); (**c**) 25 million (MD).

**Figure 7:**
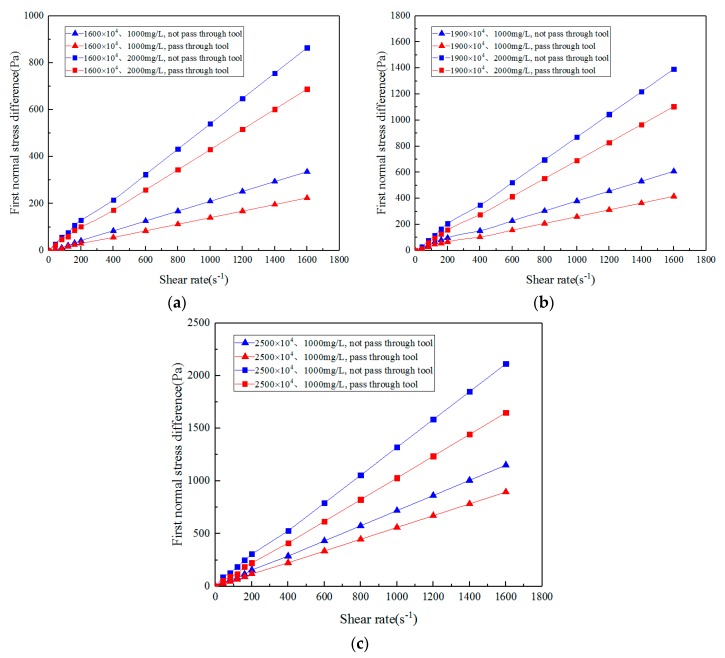
Variation of first normal stress difference with shear rate before and after ASP solution flows through the different medium tool: (**a**) 16 million (MD); (**b**) 19 million (MD); (**c**) 25 million (MD).

A steady-state shear experiment generally refers to the study of the change behavior of viscosity and elasticity of solution within a wide range of shear rates, usually including the stages when the solution structure will not be destroyed under low shear rate and will be destroyed under high shear rate. In simple steady shear flow, viscosity can be used to characterize the solution, and the first normal stress difference can be used to characterize the elasticity. In order to study the overall change trend of viscosity ignoring the unchanged viscosity under low and high shear rates, Figure 6 shows the viscosity of ASP solution with different concentrations and molecular weight as a function of shear rate and Figure 7 shows the first normal stress difference as a function of shear rate before and after the action of the different medium tool.

As shown in Figure 6, the higher the concentration and molecular weight of the solution, the higher the viscosity under the same shear rate before the solution flows through the different medium tool. With increased shear rate, the viscosity decreases, and when the shear rate reaches a certain range, the viscosity decreases gradually to a gentle level. This is because at a lower shear rate, molecular force plays a major role, molecules rely on van der Waals force interaction, and entanglement networks are formed between molecular chains. The higher the concentration of the solution, the more molecules in a unit volume, so the viscosity is greater. When the relative molecular weight of the solution is higher, the macromolecular chains are longer and it is easier for them to entangle, their conformation is more stable, and the formed reticular entangling points are firmer, so the viscosity is higher. However, with increased shear rate, the network structure among polymer molecules is destroyed (or partially destroyed) and the intermolecular force is weakened, which increases the probability of alignment consistency among molecules, reduces the relative molecular weight of the solution, and leads to decreased viscosity. When the shear rate continues to increase, the entanglement of molecular segments is stretched to the limit, and the arrangement between molecules is no longer disordered and becomes directional. At this time, the viscosity decreases gradually or remains unchanged when the shear rate increases again. On the whole, the effect of shear rate on solution viscosity is shown as shear thinning.

The viscosity curve in Figure 6 is fitted and conforms to the Carreau law, and the relaxation time λ, zero shear viscosity $\eta_{0}$ and power law index n of ASP solution before and after the action of the different medium tool are obtained by fitting, as shown in Table 3.

The higher the molecular weight and concentration of ASP solution, the greater the relaxation time value and zero shear viscosity. After the action of the different medium tool, the viscosity decreases, and power law index n after the action of the tool increases, and power law index n can represent the degree of flow characteristics deviating from Newtonian fluid, the more obvious the solution deviates from the flow characteristics of Newtonian fluid.

Bird et al. [49] described the rheological behavior of polymer solution in porous media with dumbbell molecular model, and based on this, deduced the relationship between fluid relaxation time and relative molecular mass, intrinsic viscosity and system viscosity of polymer as follows:

(16) $$\lambda =\frac{\left[\eta \right]\eta_{s}M}{AkT}$$

where [η] is the intrinsic viscosity of the solution; M is the relative molecular weight of the polymer; $\eta_{s}$ is the viscosity of the solvent; A is the Avon Gadereau constant; k is Boltzmann constant; T is the absolute temperature.

For ASP solution, the intrinsic viscosity is:

(17) $$\left[\eta \right]=\frac{\eta -\eta_{s}}{\eta_{s}c}$$

(18) $$\eta =Wc^{\alpha }M^{\beta }$$

where *W* is the system constant, *c* is the concentration of the solution, α and β are parameters that vary with the concentration. In the range of low concentration or low molecular weight, the values tends to 1, and rapidly tends to the limit value with the increase of concentration: α=5.4, β=3.4.

It can be seen from the Equations (16)–(18) that the molecular weight and concentration of ASP solution are positively correlated with the viscosity and relaxation time of the solution, so in Table 3, the influence trend of the molecular weight and concentration of ASP solution before and after the action of the different medium tool on the zero shear viscosity and relaxation time of the solution is correct.

After ASP solution flows through the different medium tool, the change trend of viscosity is similar to that before the tool, but after the tool, the viscosity decreases, because the action mechanism of the tool is to reduce the viscosity of ASP solution through shearing action. When the solution flows through the contraction section of the tool, its speed change is the most intense due to the reduced cross-sectional area forming high-strength shearing action, and the damage to the intermolecular network structure is the most serious [30], causing defects, breakage of molecular chains, rarer meshes, and reduced intermolecular force, reducing the molecular weight and viscosity [67]. Moreover, due to the effect of molecular chains and molecular structure grids, the higher the molecular weight of ASP solution, the longer the molecular chain, and the stronger the ability of molecular weights to entangle with each other, the higher the viscosity [68,69] and the stronger the shear resistance. The higher the concentration of ASP solution, the more the number of molecular chains in the solution. The molecular chains are intertwined due to insufficient extension, resulting in increased viscosity [69,70,71] and stronger shear resistance.

According to the shear test, the different medium tool can effectively reduce the viscosity of the injected ASP solution, meet the design requirements, and achieve the purpose of injecting ASP solution into low-permeability oil layers for oil displacement.

The analysis of first normal stress difference test data shows that the solution is unstable at low shear rates, which is due to the limitation of the measuring range of the instrument. In order to ensure analytical accuracy, the data of the stable section are selected for curve fitting and data analysis.

As shown in Figure 7, the greater the solution concentration and molecular weight, the greater the first normal stress difference at the same shear rate before ASP solution flows through the different medium tool. With increased shear rate, the first normal stress difference increases linearly in the stable section. This is because the higher the concentration of the solution and the larger the molecular weight, the more molecules per unit volume, the longer the molecular chains, and the stronger the mutual attraction and entanglement between molecules, which is conducive to the elastic recovery of the chain segments during deformation. With increased shear rate, the molecular unwrapping rate of ASP solution increases rapidly, while the entanglement rate changes slightly, thus making the entanglement density smaller, increasing the holes, promoting the peristalsis of molecular chains, and reducing the tendency of shearing to produce separation, thus increasing the first normal stress difference of the solution [72,73].

When ASP solution flows through the different medium tool, the first normal stress difference of the solution and the elasticity decrease. This is because before the molecular conformation is unfolded, the molecular chains are wrapped into clusters. This is the unwrapping phase, and the elasticity at this time is unwrapping elasticity. When ASP solution flows through the different medium tool, the shearing action on the molecules is strengthened, and the molecular conformation is completely expanded. At this time, the molecular chains are stretched and the elasticity is obvious, and it is called stretch elasticity. Before flowing through the different medium tool, the molecular chains of the polymer solution are long and short, with obvious size distribution and tensile elasticity of the solution. After passing through the tool, the shearing degree of the long-chain molecules is greater than that of the short-chain molecules, which makes the length distribution of the chains more uniform. Therefore, the tensile elasticity of long-chain molecules decreases significantly.

Since the first normal stress difference of ASP solution in the stable section increases linearly with the shear rate, and the higher the concentration and molecular weight of the solution, the higher the elasticity and the greater the slope of the first normal stress difference and the shear rate straight line, the slope of the first normal stress difference and the shear rate straight line (expressed by SN) can quantitatively represent the elasticity of the solution, which is convenient for comparing different solutions. The elasticity SN1 and SN2 of ASP solution with different molecular weights and concentrations before and after flowing through the different medium tool are shown in Table 4.

In this study, the linear slope of the first normal stress difference with the change of shear rate is used to quantitatively express the elasticity of the solution, which can result in a more accurate comparison. As can be seen from the table, the higher the molecular weight and concentration of ASP solution, the greater the elasticity and the greater the degree of decreased elasticity after passing through the different medium tool. At the same time, the degree of influence of molecular weight on elasticity is greater than that of concentration.

According to the results of the steady shear test, the molecular weight or concentration of the solution can be increased in actual production to improve the viscoelasticity and the oil displacement effect.

### 3.3. Effect of the Different Medium Tool on Storage Modulus and Loss Modulus of ASP Solution

**Figure 8:**
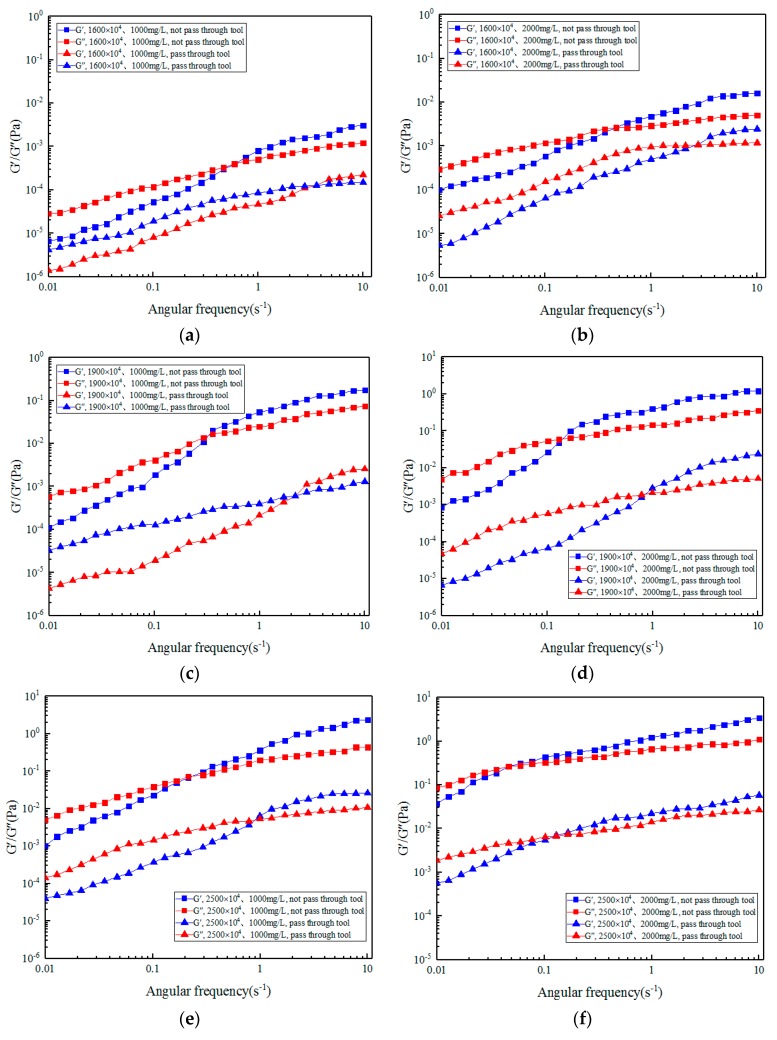
Variation of modulus with shear rate before and after ASP solution flowing through the different medium tool: (**a**) 16 million (MD), 1000 mg/L; (**b**) 16 million (MD), 2000 mg/L; (**c**) 19 million (MD), 1000 mg/L; (**d**) 19 million (MD), 2000 mg/L; (**e**) 25 million (MD), 1000 mg/L; (**f**) 25 million (MD), 1000 mg/L.

Dynamic viscoelasticity is the study of linear viscoelastic behavior of a solution, which represents the viscoelasticity of the solution without destroying its structural characteristics. In the dynamic mechanical test, the storage modulus can reflect the elasticity and the loss modulus can reflect the viscosity of viscoelastic fluid. Figure 8 shows the change of storage and loss modulus with angular frequency for ASP solution with different molecular weights and concentrations before and after the action of the different medium tool.

As can be seen from Figure 8, with increased angular frequency, the loss modulus and storage modulus of ASP solution increase before and after the action of the different medium tool, and the degree of increase of the storage modulus is greater than that of the energy dissipation modulus. Under the same angular frequency, with increased relative molecular weight and concentration of the solution, the initial values of the energy dissipation and storage modulus before and after the action of the tool are larger. Also, with increased solution concentration and molecular weight, the intersection point of storage modulus and loss modulus moves to the low-frequency direction. The storage modulus can indicate the elastic property and the loss modulus can indicate the viscous property. On the left side of the intersection point, the viscosity is greater than the elasticity, while on the right side, the elasticity is greater than the viscosity. This is because the viscosity of molecules is dominant when the deformation frequency is slow, and the elasticity is not obvious. However, when the deformation is rapid, the increased deformation energy is absorbed by the elastic deformation within or between molecules. There is not enough time for the substance to generate viscous flow, so the elasticity exceeds the viscosity. The larger the molecular weight and concentration of the solution, the more molecules in the unit volume and the longer the molecular chains, and the inner part of the formed molecular chains is wrapped into a loop, so that more instantaneous bonds formed by entanglement between molecular chains results in more spring-like properties, and the elasticity performance is much greater than the viscosity. Therefore, at low frequencies, the solution has mainly viscous flow, and at medium and high frequencies, it has mainly elastic flow. The solution with low molecular weight and low concentration has mainly viscous flow, while the solution with high molecular weight and high concentration has mainly elastic flow [74,75].

The intersection point between the storage and loss moduli is usually associated with the largest relaxation time of the solution, according to the Equations (14) and (15), the relaxation time calculated by dynamic test is shown in Table 5.

Table 5 is basically consistent with the relaxation time and zero shear viscosity in Table 3. The higher the molecular weight and concentration of ASP solution, the greater the relaxation time and zero shear viscosity. The relaxation time and zero shear viscosity decrease after the action of the different medium tool. When the relaxation time is greater than the characteristic time of oscillation test, viscous deformation will not occur and the solution will show more elasticity. When the relaxation time is less than the characteristic time of oscillation test, the solution shows more viscosity. When the relaxation time is approximately equal to the characteristic time of oscillation test, the elasticity and viscosity are equivalent, and the solution has significant viscoelasticity.

The storage modulus and loss modulus of ASP solution decrease after the solution flows through the different medium tool, but the overall change trend is consistent with that before flowing through the tool, which indicates that the viscosity and elasticity of ASP solution decrease after shearing by the tool, which is consistent with the effect of the tool on viscosity and first normal stress difference in the steady shear experiment. After the action of the different medium tool, the intersection point of the storage modulus and loss modulus curve moves to the high-frequency direction, and the viscosity property is greater than the elastic property, so the effect of the tool on viscosity is greater than the effect of elasticity, and the flow will mainly be viscous flow. With increased molecular weight and concentration of ASP solution, the intersection point of storage modulus and loss modulus curves after the action of the different medium tool moves to the low-frequency direction, then the effect of the tool on viscosity is higher than that of elasticity. This is because, at a lower shear rate, molecular force plays a major role. The higher the concentration, the more molecules per unit volume, and the stronger the attraction between molecules, so the greater the viscosity. And the larger the relative molecular weight, the greater the attraction between molecules, and the more stable the conformation of the molecular chain, so its viscosity is also larger. Viscoelasticity of ASP solution is because its molecular conformation can be changed under the action of external force (shear force under the action of the different medium tool), that is, the curled polymer chain can be stretched, and when the stretching force is removed, it can return to its natural curled shape. However, the chain segment adjusts its conformation slowly under the action of external force, so the molecular deformation lags behind the stress and shows viscoelasticity. The viscoelasticity of the solution depends on the flexibility of the molecular chain. The greater the flexibility of the chain, the more obvious the viscoelasticity.

Based on the analysis results of steady shear experiment and dynamic mechanics experiment, the effect of the different medium tool meets the design requirements. Compared with ASP solution with low molecular weight and low concentration, ASP solution with high molecular weight and high concentration has better viscoelasticity and higher oil displacement effect under the effect of the different medium tool.

## 4. Conclusions

In this paper, scanning electron microscopy, steady shear testing, and dynamic mechanical testing were used to study the molecular micromorphology of ASP solution with different molecular weights and concentrations before and after flowing through the different medium tool, and the rheological property and viscoelasticity were tested. According to the experimental results, the following conclusions were obtained:

1. The molecular chain of ASP solution is broken and the spatial grid structure is destroyed under the action of the different medium tool. The viscosity and elasticity of the solution decreased, but the effect on the viscosity was greater.
2. The elastic effect of ASP solution is stronger than the viscous effect after the action of the different medium tool.
3. The relaxation time, zero shear viscosity and other physical parameters obtained in the steady shear experiment are basically consistent with the results of the dynamic mechanical experiment, and the experimental results of the effect of the different medium tool on ASP solution are true and effective.
4. The shearing effect of the different medium tool on ASP solution with high concentration and high molecular weight (25 million, 2000 mg/L) is the best, and the oil displacement effect in low permeability oil layer is the best.

## Figures and Tables

**Table 1:** Structural parameters of the different medium tool.

Contraction Radius R_1_ (mm)	Contraction Length l_1_ (mm)	Cylinder Length l_2_ (mm)	Cylinder Radius R_2_ (mm)	Diffusion Length l_3_ (mm)	Diffusion Radius R_3_ (mm)
5	3	2	2	1	3

**Table 2:** Composition of injection and formation brine.

Chemical Composition	Na^+^	K^+^	Ca^2+^	Mg^2+^	SO_4_^2−^	Cl^−^	HCO_3_^−^
Concentration (mg/L)	853	18	54	174	270	601	186

**Table 3:** Physical parameters before and after the action of the different medium tool.

ASP Solution	Before Action of Tool $\lambda_{1}\;(s)$	After Action of Tool $\lambda_{2}\;(s)$	Before Action of Tool $\eta_{o1}$ (mPa⋅s)	After Action of Tool $\eta_{o2}$ (mPa⋅s)	Before Action of Tool $n_{1}$	After Action of Tool $n_{2}$
16 million 1000 mg/L	0.243	0.205	42.1	25.2	0.569	0.624
16 million 2000 mg/L	0.251	0.218	216.52	68.98	0.439	0.565
19 million 1000 mg/L	0.252	0.225	55.71	37.19	0.557	0.594
19 million 2000 mg/L	0.261	0.239	226.73	113.02	0.442	0.526
25 million 1000 mg/L	0.287	0.269	64.96	47.02	0.604	0.638
25 million 2000 mg/L	0.303	0.275	268.69	171.43	0.448	0.491

**Table 4:** Elastic change law of ASP solution before and after flowing through the different medium tool.

Molecular Weight (million)	Concentration (mg/L)	Before Action of Tool SN1 (Pa⋅s)	After Action of Tool SN2 (Pa⋅s)
16	1000	0.21	0.14
16	2000	0.54	0.43
19	1000	0.38	0.26
19	2000	0.87	0.69
25	1000	0.72	0.56
25	2000	1.32	1.03

**Table 5:** Relaxation time and zero shear viscosity of alkali/surfactant/polymer (ASP) solution before and after flowing through the different medium tool in dynamic test.

Molecular Weight (million)	Concentration (mg/L)	Before Action of Tool $\eta_{o1}$ (mPa⋅s)	Before Action of Tool $\eta_{o2}$ (mPa⋅s)	Before Action of Tool $\lambda_{1}\;(s)$	After Action of Tool $\lambda_{2}\;(s)$
16	1000	45.62	26.36	0.246	0.208
16	2000	219.38	68.73	0.253	0.221
19	1000	56.79	39.67	0.256	0.226
19	2000	228.98	115.49	0.268	0.243
25	1000	68.38	48.94	0.293	0.271
25	2000	272.68	176.38	0.309	0.277
